# Histopathological Analysis of *Salmonella* Chronic Carriage in the Mouse Hepatopancreatobiliary System

**DOI:** 10.1371/journal.pone.0084058

**Published:** 2013-12-12

**Authors:** Geoffrey Gonzalez-Escobedo, Krista M. D. La Perle, John S. Gunn

**Affiliations:** 1 Departments of Microbiology and Microbial Infection and Immunity, Center for Microbial Interface Biology, The Ohio State University, Columbus, Ohio, United States of America; 2 Department of Veterinary Biosciences, Comparative Pathology and Mouse Phenotyping Shared Resource, The Ohio State University, Columbus, Ohio, United States of America; Albert Einstein College of Medicine, United States of America

## Abstract

*Salmonella* Typhi asymptomatic chronic carriage represents a challenge for the diagnosis and prevention of typhoid fever in endemic areas. Such carriers are thought to be reservoirs for further spread of the disease. Gallbladder carriage has been demonstrated to be mediated by biofilm formation on gallstones and by intracellular persistence in the gallbladder epithelium of mice. In addition, both gallstones and chronic carriage have been associated with chronic inflammation and the development of gallbladder carcinoma. However, the pathogenic relationship between typhoid carriage and the development of pre-malignant and/or malignant lesions in the hepatopancreatobiliary system as well as the host-pathogen interactions occurring during chronic carriage remains unclear. In this study, we monitored the histopathological features of chronic carriage up to 1 year post-infection. Chronic cholecystitis and hepatitis ranging from mild to severe were present in infected mice regardless of the presence of gallstones. Biliary epithelial hyperplasia was observed more commonly in the gallbladder of mice with gallstones (uninfected or infected). However, pre-malignant lesions, atypical hyperplasia and metaplasia of the gallbladder and exocrine pancreas, respectively, were only associated with chronic *Salmonella* carriage. This study has implications regarding the role of *Salmonella* chronic infection and inflammation in the development of pre-malignant lesions in the epithelium of the gallbladder and pancreas that could lead to oncogenesis.

## Introduction

Typhoid or enteric fever, caused primarily by *Salmonella enterica* subsp. *enterica* serovar Typhi (*S.* Typhi), is a human systemic disease that is responsible for an estimated 21 million new infections per year resulting in approximately 200,000 deaths worldwide [1]. It is an important health problem in developing countries and poses a significant risk to travelers. After ingestion through contaminated water or food, bacteria cross the intestinal epithelial barrier, migrate into the mesenteric lymph nodes, replicate in the reticulo-endothelial system and spread systemically producing significant inflammation and acute disease [2-6] with life-threatening complications including intestinal hemorrhage and perforation, septicemia and meningitis [7-9]. During this systemic infection, *S.* Typhi can reach the gallbladder from the liver and establish an acute infection with inflammation (cholecystitis) or chronically persist in this organ. As clinical evidence, inflammation of the gallbladder and bile ducts as well as sonographic gallbladder abnormalities have been reported in acute and chronic typhoid fever patients [7,10-13]. Although hepatomegaly is encountered in approximately 30%-50% of typhoid patients with or without clinical manifestations [6,14], severe hepatic involvement concomitant with acute hepatitis is seen in 1-26% of typhoid fever patients [15] and the mortality rate due to typhoid hepatitis is reported to be between 20%-33% [16,17].

It is estimated that between 3-5% of typhoid fever patients become chronic carriers with the gallbladder being the primary site of carriage [7,18,19]. Because *S.* Typhi is a human specific pathogen, these carriers serve as a critical reservoir for further spread of the disease through bacterial shedding in feces, which is a sporadic and intermittent event [10,20]. Chronic infections can persist for decades and although highly contagious, they are typically asymptomatic, making identification of carriers within a population difficult [21,22]. 

Particularly in areas of high endemicity, the carrier state has been highly associated with pre-existing hepatobiliary disease including cholelithiasis (presence of gallstones in the gallbladder), biliary obstruction, intrahepatic cholestasis due to Caroli’s disease, biliary cirrhosis, hepatic hematoma, echinococcal cysts and amoebic abscesses [7,23]. Approximately 80-90% of chronically infected carriers have gallstones [24-28]. We have shown that *Salmonella* can form biofilms on the surface of cholesterol gallstones in the gallbladder of mice and human carriers, and this biofilm formation has been demonstrated to be a mechanism of persistence and chronic colonization in the gallbladder [29]. The biofilm state can alter the host-pathogen interaction and is often associated with a reduction of the host inflammatory response that has been referred to as a “silent chronic inflammation” [30]. 

In addition to the complications related to the acute phase of the disease, especially in the ileum and lymph organs [9], typhoid carriage complications include chronic hepatitis, acute or chronic cholecystitis, cholangitis, chronic diarrhea and rarely, pancreatitis [6,27]. However, the development of gallbladder cancer is the most severe complication associated with chronic carriage. Gallbladder cancer is the fifth most common malignancy of the gastrointestinal tract and the most common and aggressive type among the biliary tract malignancies [31]. Unfortunately, because of the delayed clinical presentation relative to pathologic progression, most of the gallbladder carcinomas are in an advanced stage when diagnosed and metastasis to the liver and regional lymph nodes are common [32]. Although infrequent in most Western countries, such cancers are highly prevalent in Chile, India and Pakistan [31,33] where there is a coincident increase in gallstone disease and typhoid fever [33,34]. 

Chronic carriers have an approximately 8-14 fold increased risk of developing gallbladder carcinoma and approximately 150-fold increased risk of developing hepatobiliary carcinoma than non-carriers [34-41]. Moreover, among patients with gallstones, the chronic typhoid carrier state was shown to be the primary independent risk factor for the development of gallbladder cancer [34]. Mortality in typhoid carriers as a result of hepatobiliary carcinomas has been reported to be between 3-6% [42]. It has been hypothesized that bacterial degradation of bile salts and chronic cholecystitis related to gallstones promotes gallbladder carcinomas [43]. In addition to gallbladder carcinomas, typhoid carrier patients have increased risks of pancreatic carcinoma [5,27,36]. 

In this study, we demonstrate that *Salmonella* not only persist in the gallbladder and liver of chronically infected mice but also cause chronic-active inflammation that can vary from mild to severe. In addition, chronically infected mice showed epithelial changes such as atypical hyperplasia/dysplasia and metaplasia in the gallbladder and pancreas as early as 3 months post-infection. Although gallstone disease and subsequent chronic inflammation are known risk factors for the development of human gallbladder and pancreas cancer [33,44,45], this is the first prospective study that describes the inflammation patterns and epithelial changes occurring during *Salmonella* chronic carriage in the absence or presence of gallstones. 

## Materials and Methods

### Ethics Statement

Mice were housed and used in strict accordance with guidelines established by The Ohio State University Institutional Animal Care and Use Committee (IACUC), and all efforts were made to minimize animal suffering. The work performed in this study was approved by the OSU IACUC. 

The Ohio State University Animal Care and Use Program is accredited by The Association for the Assessment and Accreditation of Laboratory Animal Care International (AAALAC). The protocol identification number is 2009A0057. All research activities conform to the statutes of the Animal Welfare Act and the guidelines of the Public Health Service as issued in the Guide for the Care and Use of Laboratory Animals (revised 1996).

### Bacterial strains

Wild-type strain of *S. enterica* serovar Typhimurium (*S*. Typhimurium) ATCC14028 was used in this study. Because *S.* Typhi is a human-restricted pathogen, in vivo studies of *S.* Typhi pathogenesis typically involve a mouse model of infection using *S*. Typhimurium. The pathological features of the course of mouse infection with *S*. Typhimurium are similar to those of human infection with *S.* Typhi [46].

### Mice infections and bacteria enumeration

We used a murine model of typhoid chronic infection using six-eight week old 129X1/SvJ mice (Jackson Laboratories, ME). We previously developed this animal model to study *Salmonella* chronic infections in the gallbladder that corroborated parallel human studies [29]. *S*. Typhimurium has been demonstrated to persist in the tissues of this mouse strain up to 1 year post-infection [47], but does not typically result in a lethal infection. This is due in part to the presence of a wild-type copy of the gene encoding the natural resistance-associated macrophage protein 1 (*Nramp1 or Slc11a1*). NRAMP1 is a crucial factor in controlling the replication of intracellular bacteria [48]. It exerts this role by stimulating expression of lipocalin-2, which in turn scavenges iron-loaded bacteria siderophores and mediates iron efflux from macrophages [49].

 Female 129X/SvJ mice (n=160) were fed a normal diet (Harlan laboratories, IN) (n=80) or a lithogenic diet containing 1% cholesterol (Sigma, MO) and 0.5% cholic acid (Sigma) (n=80) for nine weeks to induce cholesterol gallstones formation. Mice were inoculated intraperitoneally with 10^4^ *S*. Typhimurium or left uninfected as controls. For the remaining period, all mice were fed a normal diet (Harlan laboratories). Mice were sacrificed at 3, 6, 9 and 12 months post-infection (mpi). Thus, each time point comprised four groups (n=10 per group): uninfected mice fed a normal diet (group 1), uninfected mice fed a lithogenic diet (group 2), *Salmonella* infected mice fed a normal diet (group 3) and *Salmonella* infected mice fed a lithogenic diet (group 4). Spleen and feces from all mice of each group (n=10) and liver, pancreas, gallbladder, bile, gallstones from 3 mice of each group were homogenized and/or diluted in 1X phosphate buffered saline (PBS) for bacterial enumeration on *Salmonella*-*Shigella* agar (Difco^TM^, Becton Dickinson, MD). In the rare event when mice fed a lithogenic diet did not develop gallstones, they were excluded from the study. 

### Histopathology of the hepatopancreatobiliary system of chronically infected mice

Gallbladder, liver and pancreas (from the 7 mice that remained from each group) were fixed in 10% neutral buffered formalin phosphate (Fisher Scientific, MA) for 72 hours. Fixed tissues from groups 1 and 2 (n=3-5 each) and groups 3 and 4 (n=5 each) were randomly selected for further evaluation. Tissues were processed by routine methods and embedded in paraffin wax. Sections (4 µm) were stained with hematoxylin and eosin (HE), and evaluated with an Olympus BX45 light microscope with attached DP25 digital camera (B & B Microscopes Limited, Pittsburg, PA) by a veterinary pathologist (KMDL) certified by the American College of Veterinary Pathologists (ACVP). Inflammatory lesions were scored according to a modified grading scheme [50] (Table 1). 

**Table 1:** Histopathological scoring of the hepatopancreatobiliary system during chronic *Salmonella* carriage in mice.

**Tissue-specific inflammation**											
**Liver**				**Gallbladder**				**Pancreas**			
0	Normal			0	Normal			0	Normal		
1	Focal to multifocal portal/lobular/perivascular lymphohistiocytic ± neutrophilic infiltrates; no necrosis			1	Focal to multifocal serosal/adventitial neutrophilic ± lymphohistiocytic inflammation			1	Focal to multifocal lymphocytic aggregates limited to the interstitium/around blood vessels/within adjacent adipose tissue		
2	Multifocal to widespread portal/lobular lymphohistiocytic ± neutrophilic inflammation with necrosis of individual hepatocytes			2	Widespread transmural neutrophilic ± lymphohistiocytic inflammation			2	Focal to multifocal neutrophilic ± lymphohistiocytic inflammation with the interstitium or duct with or without lymphocytic aggregates in the interstitium/around blood vessels/within adjacent adipose tissue		
3	Multifocal to widespread portal/lobular lymphohistiocytic ± neutrophilic inflammation with focally extensive hepatocellular necrosis			3	Necrosuppurative inflammation of lumen ± wall with or without transmural neutrophilic ± lymphohistiocytic inflammation			3	Necrosuppurative inflammation obliterating ducts/vessels with or without lymphocytic aggregates in the interstitium/around blood vessels/within adjacent adipose tissue		
4	Multifocal to widespread portal/lobular lymphohistiocytic ± neutrophilic inflammation with MF coalescing areas of hepatocellular necrosis										
**Applicable to all tissues**											
**Hyalinosis**		**Bacteria visible by HE staining**				**Vascular changes**				**Atypical lymphocytic aggregates & mitotic figures**	
0	Absent	0	No			0	Absent			0	Absent
1	Focal to multifocal epithelial cytoplasmic hyaline material ± crystals; no epithelial hyperplasia	1	Yes			1	Fibrin thrombosis ± dystrophic mineralization			1	Focal
2	Multifocal epithelial cytoplasmic hyaline material ± crystals with associated epithelial hyperplasia					2	Fibrinoid vascular degeneration ± myointimal hypertrophy ± mural inflammation with or without thrombosis/dystrophic mineralization			2	Multifocal
3	Widespread to diffuse epithelial cytoplasmic hyaline material ± crystlas with associated epithelial hyperplasia										

Modified from Fadl et al. [50]

### Immunohistochemistry (IHC)

Paraffin sections from the tissues noted above were deparaffinized with xylene and graded ethanols, hydrated to distilled water and stained using the avidin-biotin complex (ABC) method by the OSU Comparative Pathology and Mouse Phenotyping Shared Resource. Briefly, sections were treated with proteinase K for 5 min, rinsed, treated with hydrogen peroxide for 10 min, rinsed and blocked with serum-free protein for 10 min, incubated for 30 min with anti-LPS *Salmonella* (1/500 dil.; Novus Biologicals, CO), and rinsed and incubated for 30 min with mouse adsorbed biotinylated rabbit anti-mouse antibody (1/1000 dil.) (Vector laboratories, CA). Samples were incubated with Vector RTU ABC Elite complex for 30 min, rinsed and incubated with chromagen (DAB) for 5 min, counterstained with hematoxylin, rinsed and treated with 1% ammonium hydroxide, dehydrated in ethanol, cleared in xylene and mounted on coverslips. 

### Statistical Analysis

Student’s *t*-test analysis was performed to detect statistically significant differences between means of bacteria enumeration and of histology scores (p<0.5). This was performed by comparison between experimental groups (± infected, ± gallstones) and between time points. 

## Results

### *Salmonella* was recovered at all time points from the feces and spleens of mice without gallstones, but inconsistently observed in other tissues/fluids.

**Figure 1:**
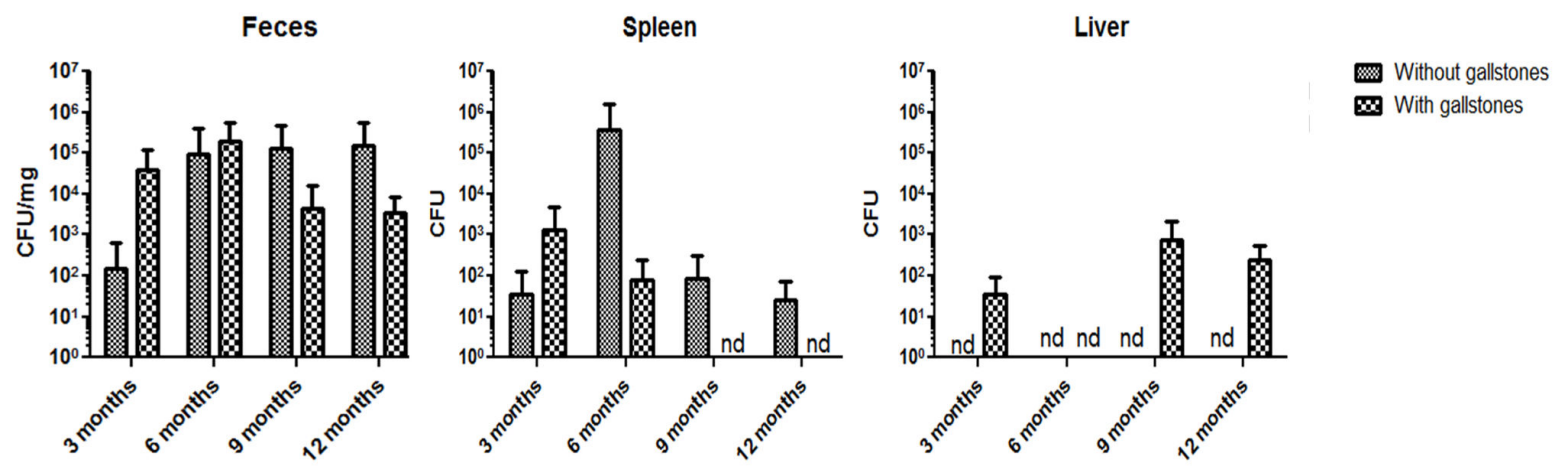
*Salmonella* detection in organs and bodily fluids of chronically infected mice up to 1 year post-infection. Bacterial CFU enumeration of *Salmonella* at various times post-infection in the absence and presence of gallstones. nd, not detected. Limit of detection was 100 CFU /mL. No statistically significant difference was observed between infected mice with or without gallstones at any time point (Student’s *t* test). nd, not detected.

In previous work by our laboratory using 129X1/SvJ mice, we consistently observed *Salmonella* in all of the tissues/fluids examined in this study up to at least 21 days post infection [29]. However, *Salmonella* long-term colonization in the hepatopancreatobililary system in the absence or presence of gallstones has not been previously determined. In order to document the bacterial burden up to 1 year post-infection, bacterial enumeration in feces and spleen was performed for all mice (n = 10/group). *Salmonella* was recovered from the feces of all infected mice at all time points. The spleen was colonized at all time points except in infected mice with gallstones at 9 and 12 mpi (Figure 1). CFU enumeration for the remaining tissues was performed for only 3 mice per group. In the liver, bacteria were recovered in some time points but only from mice harboring gallstones (Figure 1). *Salmonella* was infrequently recovered from the pancreas, gallbladder, bile and gallstones (data not shown). It is important to note that the CFU limit of detection was 100 CFU/mL. 

### *Salmonella* was detected by IHC in the liver, gallbladder and pancreas of some infected mice.

**Figure 2:**
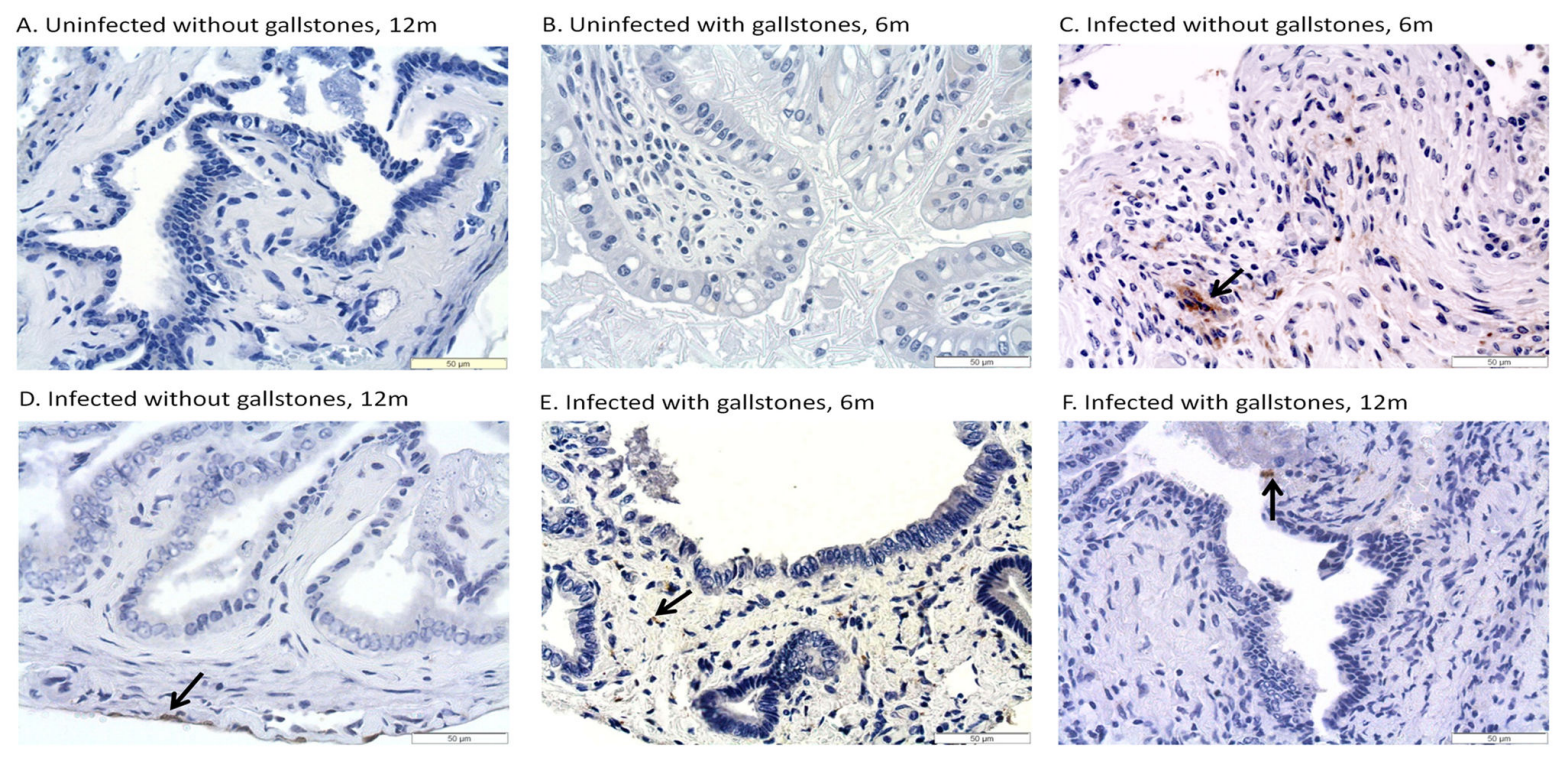
*Salmonella* LPS staining was frequently detected in the gallbladder of infected mice regardless of the presence of gallstones. Representative IHC images of mouse gallbladder tissue at 6 and 12 months post-infection using an anti-*Salmonella* LPS antibody. Such bacterial staining occurs at time points in which no CFU were detected from the gallbladder of cage mates. Black arrows show *Salmonella* LPS staining in the muscularis (Panel C and D), in the epithelium (Panel E) or in the lumen (Panel F). DAB and hematoxylin counterstain. 40x.

**Figure 3:**
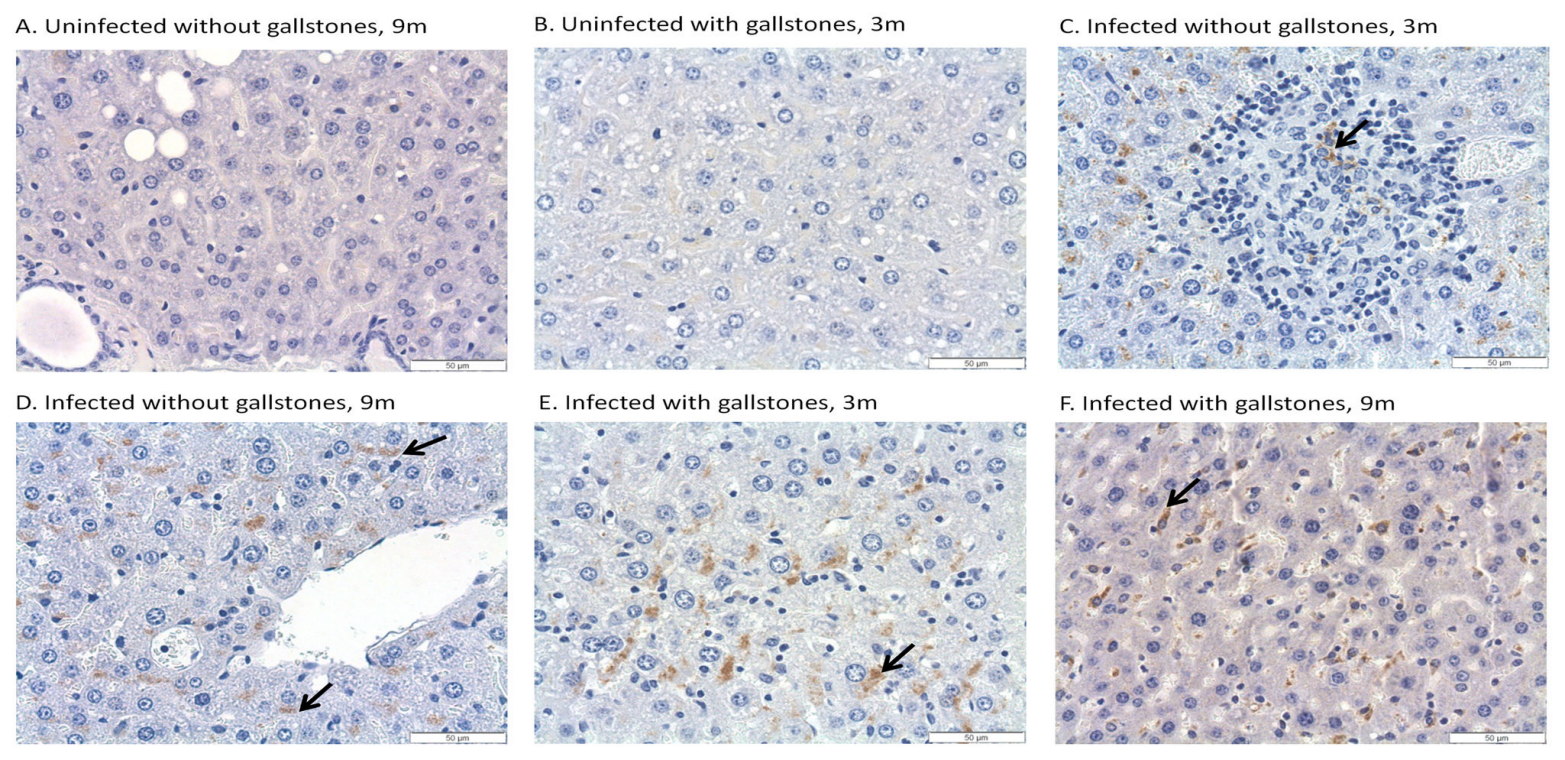
*Salmonella* LPS staining was detected in the liver regardless of the presence of gallstones. Representative IHC images of mouse liver sections at 3 and 9 months post-infection using an anti-*Salmonella* LPS antibody. Panel C shows a typhoid nodule. Black arrows indicate the presence of *Salmonella* inside inflammatory macrophages (Panel C) or Kupffer cells (Panel F) or intracellularly in hepatocytes (Panels D, E). DAB and hematoxylin counterstain. 40x.

**Figure 4:**
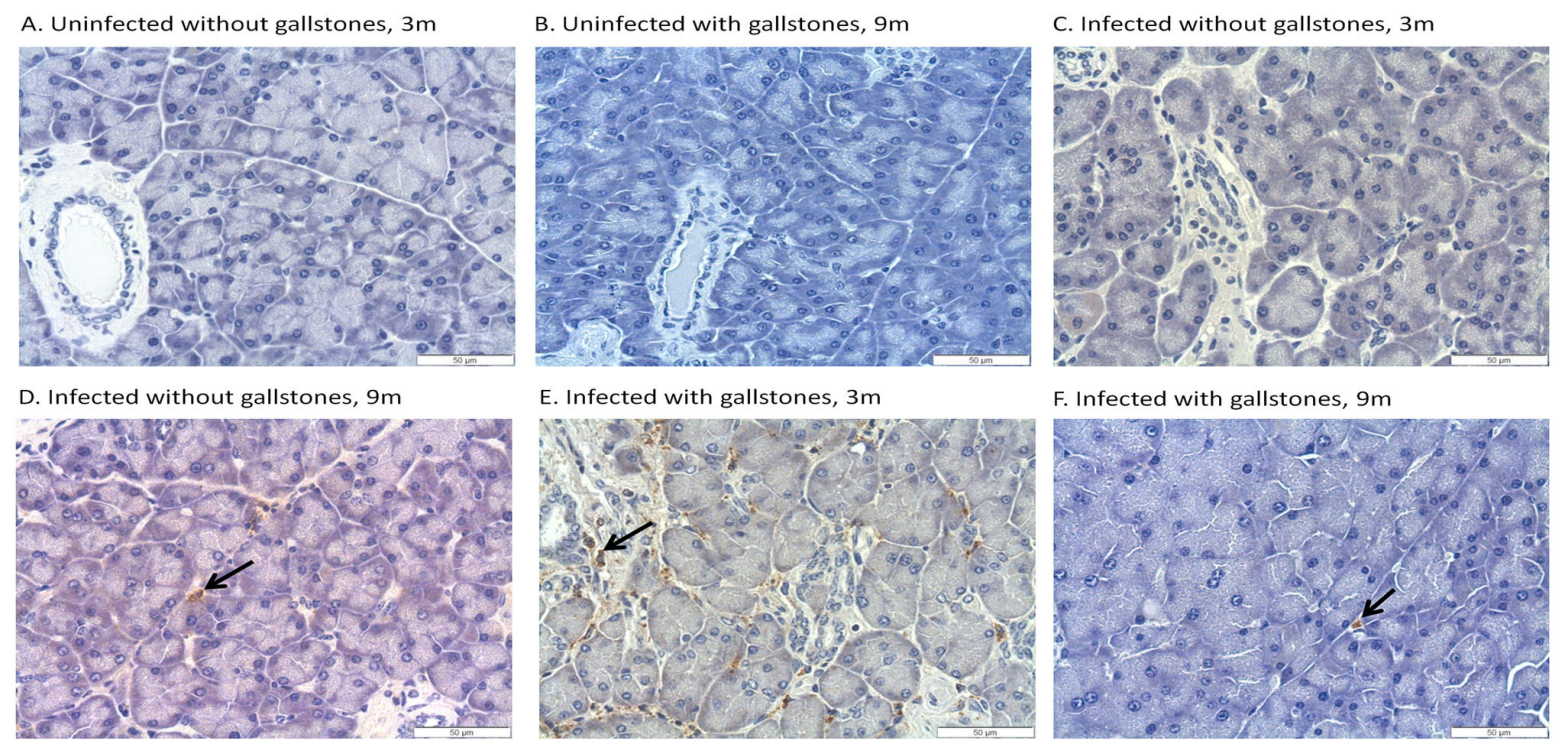
*Salmonella* LPS staining was detected in the exocrine pancreas regardless of the presence of gallstones. Representative IHC images of mouse pancreas sections at 3 and 9 months post-infection using an anti-*Salmonella* LPS antibody. *Salmonella* was detected in the interstitium around acini or ductular cells (Panels D, E and F). DAB and hematoxylin counterstain. 40x.

CFU enumeration has been shown to be a suboptimal method for *Salmonella* detection during chronic carriage [51]. Thus, to better determine the number and location of *Salmonella* in the hepatopancreatobiliary system, we detected *Salmonella* by IHC using anti-*Salmonella* LPS antibody. In the gallbladder, *Salmonella* LPS staining was detected in some of the infected mice without gallstones at 6 mpi (4 out of 5 mice tested), 9 mpi and 12 mpi (2 out of 5) whereas in infected mice with gallstones it was detected at 3 mpi (3 out of 5), 6 mpi, 9 mpi and 12 mpi (2 out of 5). The localization of *Salmonella* LPS staining in the gallbladder was confined to the lumen, mucosa or muscularis (Figure 2). In the liver, *Salmonella* LPS staining was detected in infected mice without gallstones at 3 mpi and 6 mpi (5 out of 5), 9 mpi (4 out of 5) and 12 mpi (3 out of 5) whereas in infected mice with gallstones it was detected at 3 mpi (5 out of 5) and at 6 mpi, 9 mpi and 12 mpi (4 out of 5). In the liver, *Salmonella* LPS staining was observed intracellularly in macrophages associated with typhoid nodules as well as kupffer cells and hepatocytes. Interestingly, in the hepatocytes, *Salmonella* LPS was mostly detected eccentrically shifted to one side of the cytoplasm, presumably closer to the bile canaliculi invaginations (Figure 3). In the pancreas, *Salmonella* LPS staining was also detected in infected mice without gallstones at 3 mpi (1 out of 5), 6 mpi (5 out of 5), 9 mpi (3 out of 5) and 12 mpi (4 out of 5) and in infected mice with gallstones at 3 mpi (3 out of 5), 6 mpi (5 out of 5), 9 mpi (3 out of 5) and 12 mpi (2 out of 5). *Salmonella* LPS staining was mostly detected in the interstitium of the exocrine pancreas (Figure 4). The uninfected controls did not show positive reaction with the antibody.

### Chronic cholecystitis and hepatitis are the hallmark during *Salmonella* carriage

**Figure 5:**
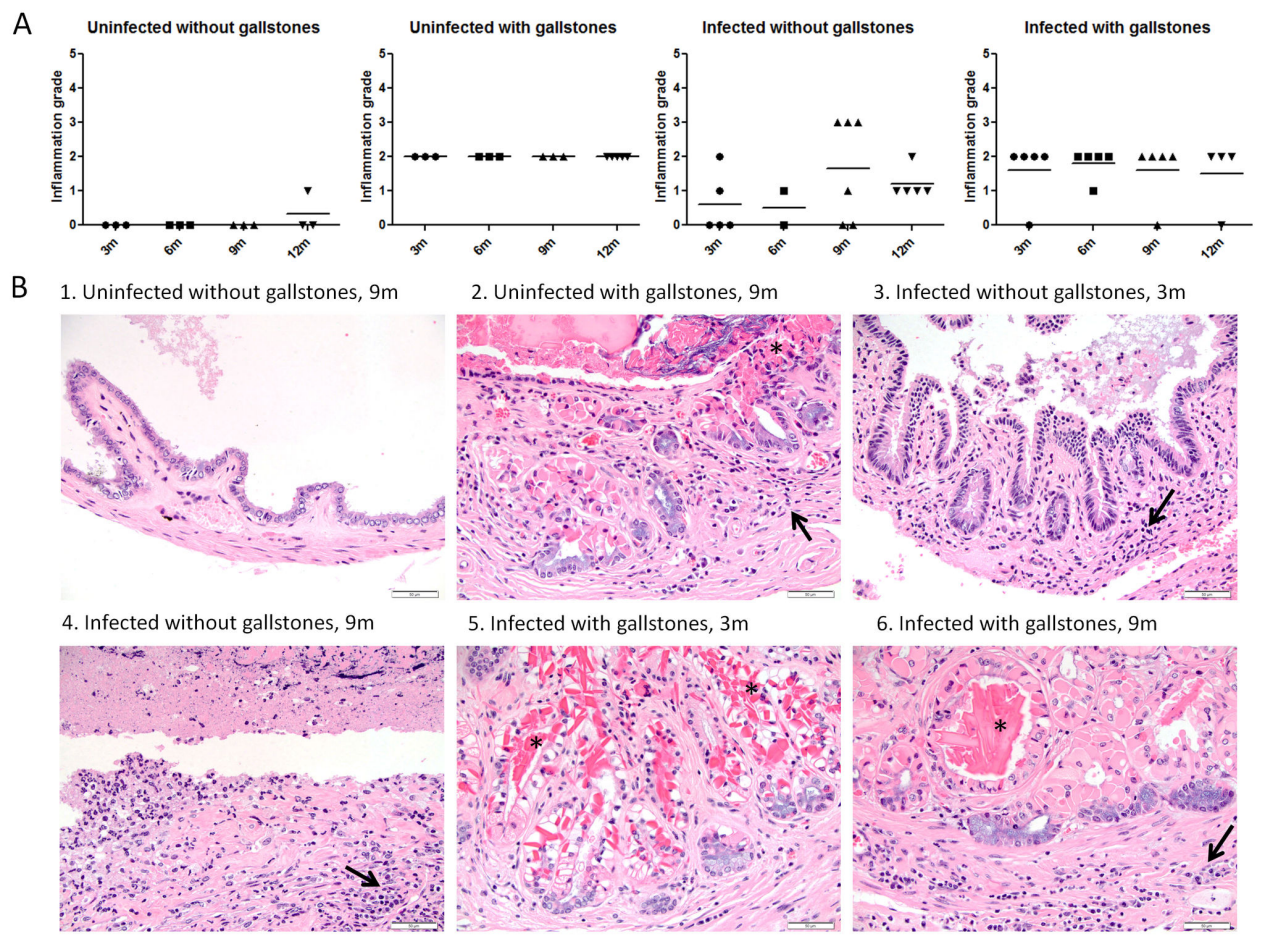
Chronic cholecystitis occurs as a result of both gallstone disease and *Salmonella* carriage. (A) Inflammation grades in the gallbladder at all time points post-infection (± bacteria, ± gallstones). Means of each time point values were compared by a Student’s *t* test. No statistical significant difference was observed between values of each time point (*p*<0.5). (B) Representative HE images of each group at 3 and 9 months post-infection. B1 has an inflammation score of 0; B2, B3, B5 and B6 have a inflammation score of 2; whereas B4 has a score of 3. Hyperplasia was only evident in mice harboring gallstones and with concurrent epithelial hyalinosis (*) (B2, B5 and B6). Black arrows indicate scattered inflammatory cells within the lamina propria and muscularis, they were mainly composed of lymphocytes, plasma cells and fewer neutrophils. 40x.

Although chronic inflammation in the liver and gallbladder has been reported in typhoid carriers, the histopathological features of carriage in the absence or presence of gallstones has not been previously assessed. Thus, we monitored the inflammation patterns in the hepatopancreatobiliary system during chronic carriage by histopathology. In the gallbladder, inflammation was multifocal, widespread, often transmural and characterized by lymphocytes, macrophages (histiocytes), plasma cells and fewer neutrophils. In general, uninfected gall bladders with gallstones exhibited more inflammation than infected gallbladders without gallstones. However, at 9 mpi, 3 infected mice without gallstones showed necrosuppurative inflammation with ulceration of the biliary epithelium (Figure 5). Mice with gallstones (+/- *Salmonella*) did not show any significant difference in their inflammation scores (p<0.5), suggesting that, when gallstones are present, *Salmonella* did not exacerbate the inflammation levels. Additionally, inflammation levels between infected mice +/- gallstones were statistically significant at 6 mpi (p<0.5) whereas inflammation levels between uninfected mice with gallstones and infected mice without gallstones were statistically significant at 6 and 12 mpi (p<0.5 and p<0.01; respectively), with inflammation higher in the former. This suggests that at these specific time points of chronic carriage, *Salmonella* did not intensify the inflammatory response in the gallbladder above the level of inflammation resulting from the mere presence of gallstones. 

**Figure 6:**
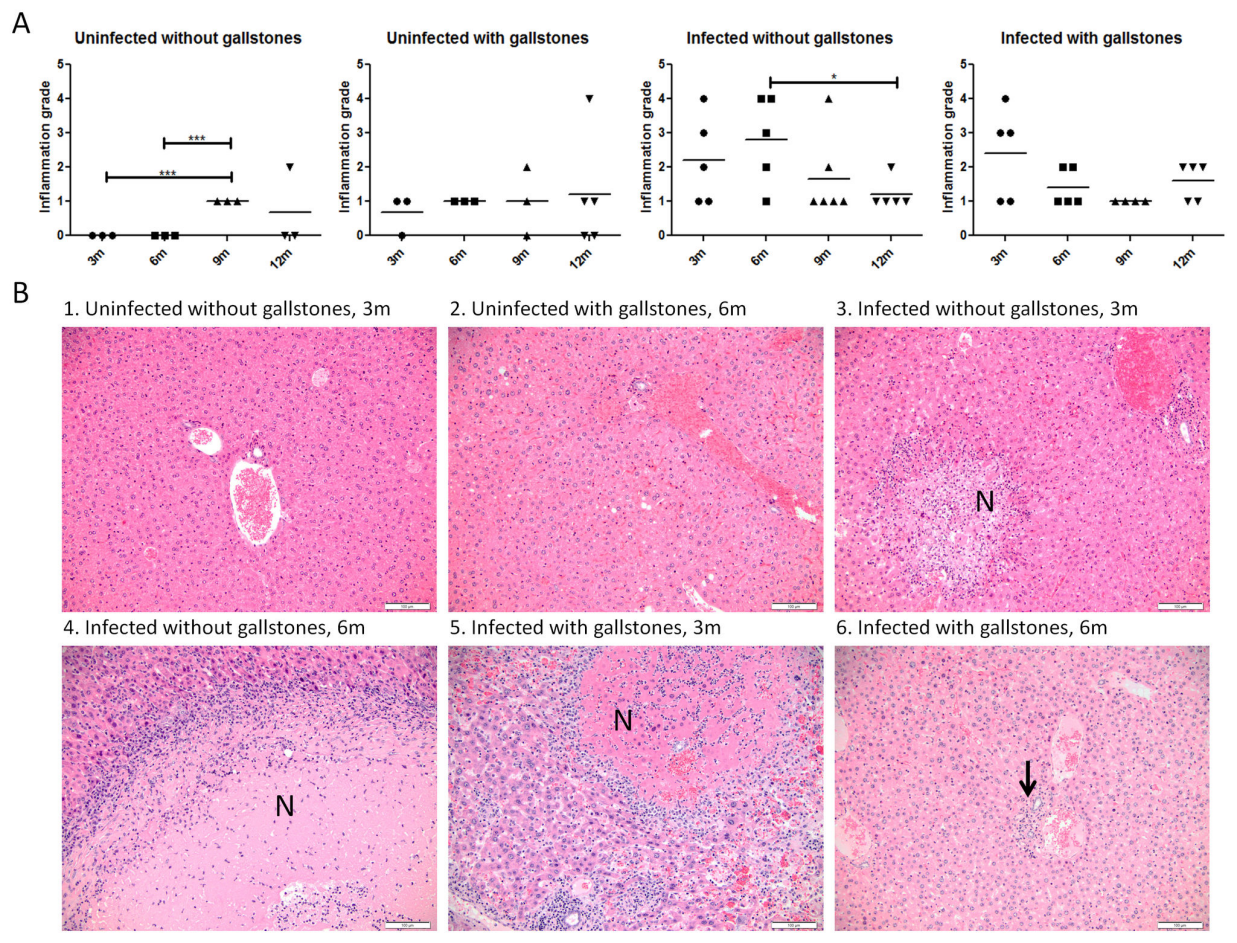
Chronic hepatitis is exacerbated in infected mice at 3 and 6 months post-infection. (A) Inflammation grades in the liver at all time points post-infection (± bacteria, ± gallstones). Means of each time point values were compared by a Student’s *t* test (*, *p*<0.05; ***, *p*<0.001). (B) Representative HE images of each group at 3 and 6 months post-infection. Inflammation scores are as follows: B1 (0); B2 (1); B3 and B6 (2); B4 and B5 (4). Infected mice have scattered inflammatory cells distributed randomly within the lobule (B3-B5) and/or in portal areas (B6) composed of neutrophils, lymphocytes, plasma cells and histiocytes. Inflammation was often associated with necrosis of individual hepatocytes (B3) or large confluent areas of hepatocellular necrosis (B4-B5) (noted as N). 20x.

 In the liver, the highest inflammation scores were present in infected mice without gallstones at 6 mpi and in infected mice with gallstones at 3 mpi (Figure 6A). Inflammatory cells were distributed randomly within the lobule and/or in portal areas. Inflammation was often associated with necrosis of individual hepatocytes or large confluent areas of hepatocellular necrosis (Figure 6B). In contrast to the gallbladder, uninfected mice without gallstones only showed mild degrees of inflammation. Classical histiocytic (macrophage-mediated) inflammation consistent with typhoid nodules was noted in the liver lobules of many infected mice regardless of the presence of gallstones (Figure 3C). 

**Figure 7:**
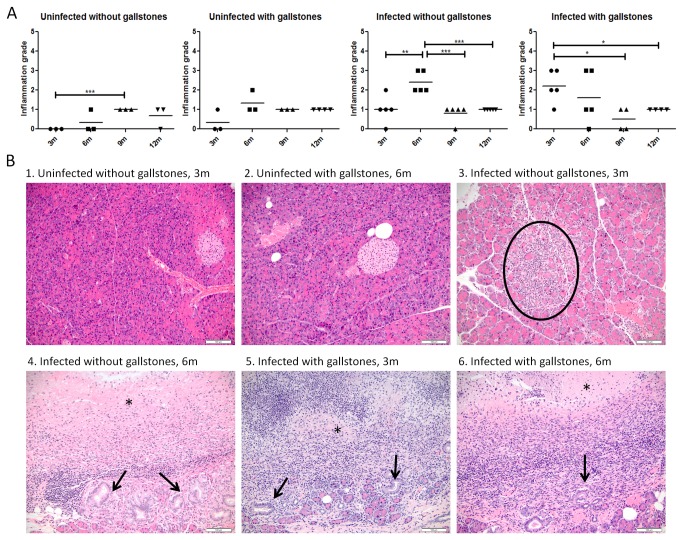
Chronic pancreatitis is exacerbated in infected mice at 3 and 6 months post-infection. (A) Inflammation grades in the pancreas at all time points post-infection (± bacteria, ± gallstones). Means of each time point values were compared by a Student’s *t* test (*, *p*<0.05; **, *p*<0.01 ***, *p*<0.001). (B) Representative HE images of each group at 3 and 6 months post-infection. Inflammation scores are as follows: B1 (0); B2 (1); B3 (2); B4, B5 and B6 (3). Inflammatory cells composed of scattered neutrophils, lymphocytes and plasma cells were occasionally distributed within the interstitium of the exocrine pancreas between acini (circle). In more severe cases, inflammation was centered upon and typically obliterated large ducts (B4-B6) and was characterized by necrotic debris (*) centrally, and lymphoplasmacytic aggregates and mucous metaplasia (black arrows) peripherally. 20x.

 In the pancreas, the inflammation patterns were similar to the liver with the highest inflammation also observed in infected mice without gallstones at 6mpi and in infected mice with gallstones at 3 mpi (Figure 7). However, after 6 mpi, the inflammation was milder in all infected mice. Inflammatory cells were mostly observed within the interstitium of the exocrine pancreas between acini. In more severe cases, inflammation obliterated large ducts and was characterized by necrotic debris (centrally), lymphoplasmacytic aggregates and mucous metaplasia at the periphery.

 Chronic vascular thrombosis with dystrophic mineralization was only observed in infected mice (with or without gallstones). In the gallbladder, these vascular changes predominantly occurred in infected mice with gallstones at 3 mpi (80%). In the liver, chronic thrombosis was only observed at 9 mpi in infected mice without gallstones (40%) and in infected mice with gallstones (100%) (Figure S1). In the pancreas, chronic arteritis was also only observed in infected mice but in less than 40% of mice per time point.

### Biliary epithelial hyperplasia was observed as a result of gallstone disease and *Salmonella* carriage

In addition to inflammation, we also monitored hyperplastic changes in the hepatopancreatobiliary organs. Biliary epithelial hyperplasia concurrent with hyalinosis was observed in the liver and gallbladder. In the gallbladder, biliary hyperplasia was observed more often in mice with gallstones (uninfected or infected) in 18/23 (78%) of the mice (Figure 5B). In the liver, hyperplasia was more common in infected mice with gallstones at 9 mpi (3 out of 4). The pancreas did not show signs of hyperplasia or hyalinosis.

### Atypical hyperplasia/dysplasia was only observed in the gallbladder of infected mice

**Figure 8:**
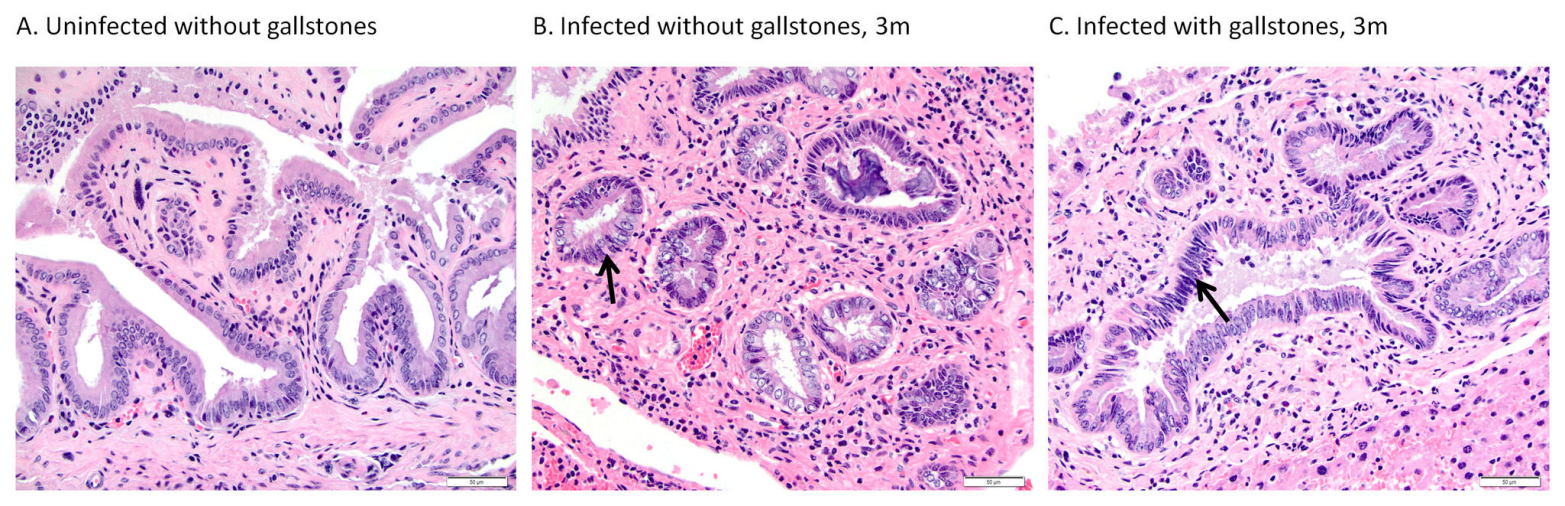
Atypical hyperplasia in the absence of marked necrosuppurative inflammation and/or epithelial hyalinosis was observed in the gallbladder of infected mice, regardless of the presence of gallstones. Representative HE images of gallbladder 3 months post-infection. Black arrows indicate tall columnar biliary epithelial cells with large crowded, basophilic to vesicular nuclei and loss of polarity.40x.

Pre-malignant lesions such as dysplasia can predict possible complications such as neoplasia of the gallbladder epithelium which has been associated with chronic *Salmonella* carriage. Atypical hyperplasia in the absence of marked necrosuppurative inflammation and/or epithelial hyalinosis was observed in the gallbladder of infected mice at 3 mpi, but not observed at later time points (Figure 8). None of these pre-malignant changes were observed in uninfected animals (with or without gallstones).

### Pancreatic mucinous metaplasia was only evident in infected mice regardless of the presence of gallstones

**Figure 9:**
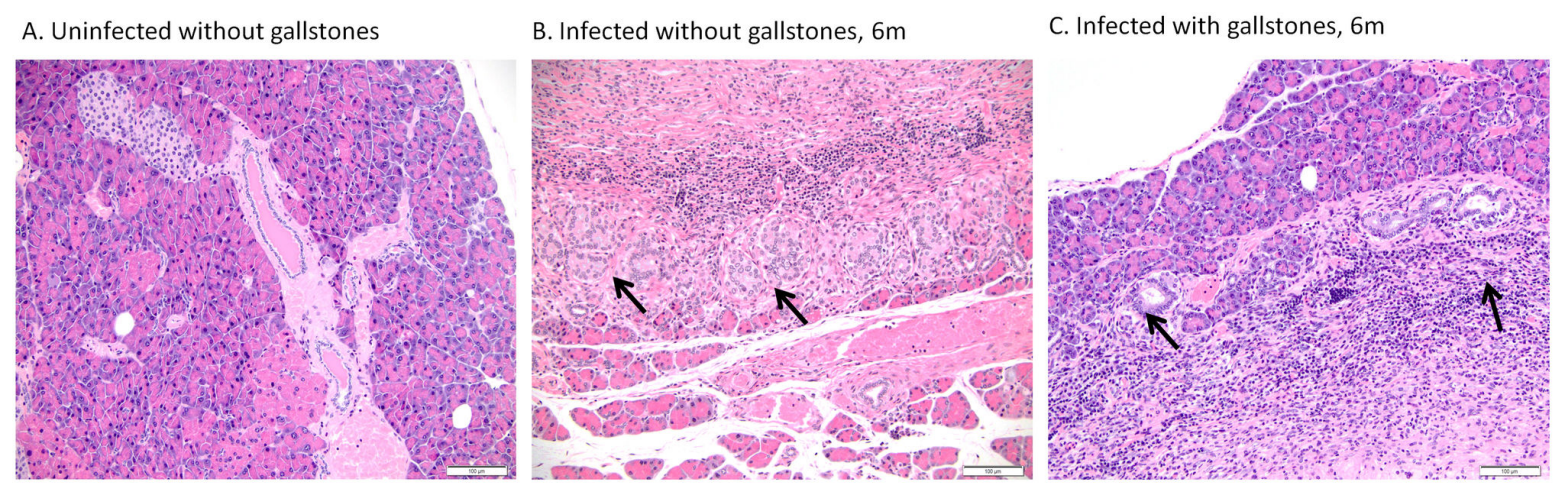
Pancreatic mucinous metaplasia was only evident in infected mice regardless of the presence of gallstones. Representative HE images of pancreas 6 months post-infection. Black arrows indicate mucinous metaplasia associated with exocrine pancreatic acini at the periphery of marked chronic necrosuppurative inflammation. 20x.

In addition to dysplasia, other pre-malignant lesions, such as metaplasia, were also monitored in *Salmonella* chronic carriage. Histopathological evaluation of the exocrine pancreas revealed mucinous metaplasia (with columnar epithelium overtly producing mucus). When present, the mucinous metaplasia was located at the periphery of marked necrosuppurative inflammation in infected mice at 3 and 6 mpi with or without gallstones. Neither the gallbladder nor the liver showed metaplasia at any time point (Figure 9). 

## Discussion

 Despite the many complications that result from chronic carriage, the host and the bacterial contributions that create the environment that allows this chronic infection as a result of *Salmonella* infection and subsequent cancers are unknown. We hypothesized that chronic inflammation in the hepatopancreatobiliary system leads to pre-malignant transformation of the gallbladder and pancreas epithelium including hyperplasia, metaplasia and dysplasia. These epithelial changes, especially metaplasia and dysplasia can be associated with chronic inflammation and they could progress to neoplasia (tumor) development [52]. It is believed that metaplasia serves as the precursor of carcinogenetic transformation, progressing into dysplasia, and culminating in invasive gallbladder carcinoma [53]. Understanding the factors that allow bacterial colonization and carriage, as well as the establishment of a mouse model to examine the contribution of carriage to oncogenesis, could lead to the development of new therapies for patients suffering from *Salmonella*-mediated chronic gallbladder disease, decreasing the incidence of typhoid fever and gallbladder cancer.

 In this study, we monitored *Salmonella* chronic colonization and the histopathological changes occurring in the hepatopancreatobiliary system up to 1 year post-infection. This is the first prospective study that compares *Salmonella* chronic carriage histopathology among infected subjects with or without gallstones. Cholelithiasis is a predisposing factor for *Salmonella* carriage and chronic inflammation, but it is also a risk factor for the development of gallbladder cancer [33,44,54,55]. Due to the likely multifactorial etiology of gallbladder cancer, the specific signatures during progression from typhoid carriage to pre-malignant lesions in the gallbladder and other hepatopancreatobiliary organs need to be distinguished from other causes and from non-malignant conditions. 

 Chronic colonization of 129X1/SvJ mice has been previously assessed up to 1 year post-infection [47]. We also evaluated *Salmonella* chronic colonization in this mouse model, but with the addition of gallstones. Bacterial enumeration showed that feces and spleen were colonized by *Salmonella* in the absence of gallstones at all time points. Interestingly, while *Salmonella* was present in feces of mice with gallstones at all time points, we did not recover *Salmonella* after 9 mpi from the spleen of infected mice harboring gallstones. The cause of this is unclear, but might be related to the immune response during prolonged infection.

 In the liver, *Salmonella* was recovered at 3, 9 and 12 mpi but only in mice harboring gallstones. Colonization was minimal in the pancreas, gallbladder and bile. However, it is important to note that only 3 mice per time point were used for enumeration (CFU) in liver, pancreas, gallbladder, bile and gallstones. It is possible that other mice in the group could have been colonized or that the *Salmonella* burden in these organs was below the limit of detection (100 CFU/mL). In fact, *Salmonella* (alive or dead) was detected by IHC in the liver, gallbladder and pancreas at time points that were negative by CFU analysis. In the gallbladder, *Salmonella* LPS staining was mostly detected in the muscularis. This is at odds to other studies performed during acute stages where *Salmonella* localization was restricted to the mucosa [56,57]. In the liver, we detected *Salmonella* LPS staining both intracellularly in inflammatory macrophages, Kupffer cells and in hepatocytes. Previous studies demonstrated that *Salmonella* primarily replicates in vivo in mouse macrophages at early stages of infection [58,59]. Thus, it is possible that hepatocytes can be colonized later during chronic stages of infection. In the pancreas, localization of *Salmonella* LPS staining was mostly in the interstitium, also suggesting that *Salmonella* resided inside macrophages, although bacteria could have also been phagocytosed and cleared.

 In previous studies, we showed that acute hepatitis and cholecystitis (neutrophilic and histiocytic) occurred up to 2 months post-infection [51]. Sonographic findings including mucosal irregularities, edema with distention and thickening of the gallbladder, suggesting cholecystitis, have been reported in acute typhoid fever patients without gallstones [12]. In fact, histiocytic inflammation and granuloma formation occurs in the gallbladder and liver during typhoid fever [27]. In this study, chronic cholecystitis and hepatitis (mostly lymphohistiocytic and plasmacytic) were the predominant findings during *Salmonella* carriage up to 1 year post-infection, which has also been reported in human typhoid carriers [23,28,60,61]. 

 Corroborating previous studies using mouse models fed a lithogenic diet [62-64], the mere presence of gallstones caused chronic cholecystitis, in general causing more inflammation than the presence of *Salmonella*. This suggests that in most cases, *Salmonella* chronic colonization does not induce strong infiltration of immune cells. However, we observed severe inflammation (necrosuppurative) in some infected mice but without gallstones, implying that this is a heterogenous process. Interestingly, the levels of inflammation were not exacerbated in the gallbladder of infected mice with gallstones, suggesting that *Salmonella* does not dramatically increase pre-existing inflammation. 

 In the liver and pancreas, the inflammation patterns were different than in the gallbladder because here, the presence of *Salmonella* alone caused more inflammation than the presence of gallstones alone (especially at 3 and 6 mpi.). This is an expected observation considering that gallstones are typically present in the gallbladder and bile ducts, and only infrequently found in the pancreatic duct. One of the pathological observations commonly seen in the liver of infected mice was the presence of focal/multifocal necrosis and typhoid nodules, which are reported pathological features of typhoid fever along with infrequent focal granulomas in the liver and spleen [15,65,66]. Typhoid nodules are primarily aggregates of altered macrophages that phagocytose bacteria, erythrocytes and degenerated lymphocytes, sometimes with central necrosis. They can also contain plasma cells and lymphocytes and atypically, neutrophils [14,65]. Since these nodules are mainly comprised of altered macrophages, this may indicate that histiocytic inflammation occurred early during infection (before 3 months post-infection in this model). In addition, in the liver we also observed mineralized chronic vascular thrombi. Venous thrombosis is a consequence of severe cases of typhoid fever [15]. 

 Acute and chronic pancreatitis, in some cases with abscesses, has been reported in typhoid fever patients [27,67-70]. In this study, we observed lymphohistiocytic and necrosuppurative inflammation in the pancreas of infected mice at 3 and 6 mpi. In comparison with the gallbladder and liver, chronic inflammation in the pancreas was milder after 6mpi.

 Chronic inflammation with oxidative stress caused by persistent bacterial infection and production of bacterial toxins and metabolites has been associated with biliary carcinogenesis [71]. At the molecular level, it has been proposed that chronic inflammation of the gallbladder leads to an allele-specific mutation with loss of p53 gene heterozygosity and overexpression of p53 protein [72,73]. This mutation is considered to result in malignant transformation of the gallbladder mucosa because p53 is a recognized tumor suppressor [74]. In addition, cyclooxygenase 2 (COX-2), which is also associated with gallbladder carcinomas, is also induced during gallbladder inflammation [44,75]. Thus, chronic inflammation can lead to molecular disturbances in the cell cycle of the gallbladder mucosa that contribute to gallbladder cancer development [45].

 Hyperplastic changes in the epithelium of the gallbladder and bile ducts of the liver were frequently noted in mice harboring gallstones, independent of *Salmonella* infection. This increased cell proliferation as a result of cholelithiasis has been previously reported [76,77]. However, typically this was seen concurrently with hyalinosis in affected tissues and/or inflammation. Hyalinosis is a common lesion in some strains of mice whereby epithelial cells in various tissues including the nasal cavity, lung, glandular stomach, gallbladder, bile and pancreatic ducts, and ureter accumulate intensely eosinophilic cytoplasm that may also be associated with spicular eosinophilic crystals. It is thought that crystals form due to local increased concentrations of the chitinase-like protein Chi3l3 (formerly Ym-1/Ym-2) secondary to neutrophil degranulation during repeated episodes of inflammation. The crystals are resistant to degradation, and the inflammation and crystals are sufficient in and of themselves to induce epithelial hyperplasia [78,79]. Biliary hyperplasia in the liver was observed more often in infected mice with gallstones at 9 mpi.

 Dysplasia is a pre-malignant lesion that can progress to neoplasia. In fact, more than 80% of invasive gallbladder carcinomas have adjacent areas of dysplasia [53]. Atypical hyperplasia/dysplasia not associated with chronic necrosuppurative inflammation or epithelial hyalinosis was observed in the gallbladder of infected mice regardless of the presence of gallstones only at 3 mpi. In fact, this is the first time that atypical hyperplasia/dysplasia has been shown as a result of *Salmonella* chronic infection. It is widely reported that gallstones are the major risk factor for developing gallbladder cancer [80]. We hypothesized that chronic inflammation of the gallbladder or bile ducts as a result of *Salmonella* infection may also lead to pre-malignant lesions or carcinogenesis. Considering that these proliferative changes are more likely to be a physiological response to inflammation and that we did not see atypical hyperplasia/dysplasia after 3 mpi, we cannot predict if these changes ultimately progress to neoplasia in these mice. However, this could be due to scarce long term (>3 months) *Salmonella* colonization of the gallbladder in our model under the chosen experimental conditions. Thus, we believe that in human chronic carriers with permanent bacterial colonization in the gallbladder, chronic inflammation and dysplasia could occur and progress to neoplasia. 

 In addition to the epithelial changes observed in the gallbladder, acinar-ductal mucinous metaplasia was observed in the pancreas (and only the pancreas) of infected mice. Acinar-ductal mucinous metaplasia occurred adjacent to acini at the periphery of necrosuppurative inflammation; morphologically, it is commonly identified as mucinous metaplastic epithelia mixed with acinar cells in the pancreatic lobules. This type of metaplasia is commonly seen in chronic pancreatitis and in the pancreatic parenchyma adjacent to ductal carcinoma [81]. The severe inflammation observed in the pancreas of infected mice seemed to originate within the ducts but then obliterated the ducts and extended into the acini. Mucinous metaplasia is likely an attempt to regenerate the duct but the acinar cells are also irritated from the inflammation. Although *Salmonella* was usually detected in the liver during chronic disease, this persistence did not induce epithelial changes such as atypical hyperplasia/dysplasia and metaplasia that were observed in the gallbladder and pancreas, respectively.

 In conclusion, chronic inflammation in organs of the hepatopancreatobiliary system was a predominant observation in our model of chronic carriage. Inflammation patterns differed in the gallbladder compared with the liver and pancreas. In the gallbladder, the presence of gallstones caused more robust and ongoing inflammation than the mere presence of *Salmonella*. However, in the liver and pancreas, the presence of *Salmonella* caused more inflammation. Interestingly, in all organs, infected mice with gallstones did not show increased inflammation in comparison with the other groups (except in the pancreas at 3 mpi.). This inflammation could explain the epithelial changes observed in the pancreas (mucinous metaplasia). In contrast, the atypical hyperplasia observed in the gallbladder was not associated with inflammation but this could have happened before 3 mpi. Although the mere presence of gallstones did not cause these epithelial changes, the low level of *Salmonella* colonization of these organs months post-infection could explain the absence of more dysplastic changes that could better correlate chronic carriage with oncogenesis. In the future, we will attempt to alter the model system to permit longer chronic hepatopancreatobiliary colonization, allowing for histological studies that likely better correlate with changes that occur during long-term human chronic typhoid carriage. 

## Supporting Information

Figure S1 **Dystrophic mineralization of venous fibrin thrombi was only present in the gallbladder and liver of infected mice with gallstones** Representative HE liver (A, C) and gallbladder adjacent to the liver (B) at 3 and 9 months post-infection, respectively. Note the mineralized thrombi in Panels B and C surrounded by portal aggregates of lymphocytes, plasma cells and neutrophils. 20x.
(TIF)
