# Supplementary figures and images for: Histopathological Analysis of Salmonella Chronic Carriage in the Mouse Hepatopancreatobiliary System
Source: PLoS One. 2013 Dec 12;8(12):e84058. doi: 10.1371/journal.pone.0084058 (PMC3861519; doi:10.1371/journal.pone.0084058)

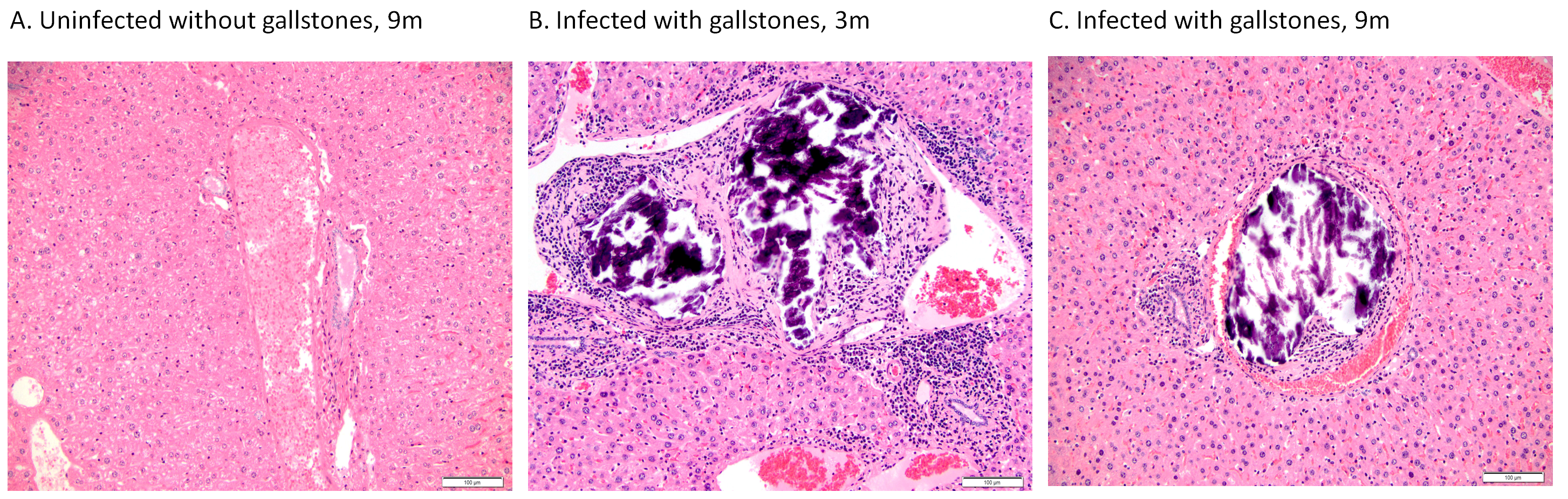

Supplement: Figure S1 — Dystrophic mineralization of venous fibrin thrombi was only present in the gallbladder and liver of infected mice with gallstones Representative HE liver (A, C) and gallbladder adjacent to the liver (B) at 3 and 9 months post-infection, respectively. Note the mineralized thrombi in Panels B and C surrounded by portal aggregates of lymphocytes, plasma cells and neutrophils. 20x. (TIF) [file pone.0084058.s001.tif]
